# Artificial Caries Lesion Characteristics after Secondary Demineralization with Theobromine-Containing Protocol

**DOI:** 10.3390/molecules26020300

**Published:** 2021-01-08

**Authors:** Hani M. Nassar, Frank Lippert

**Affiliations:** 1 Department of Restorative Dentistry, Faculty of Dentistry, King Abdulaziz University, P.O. Box 80209, Jeddah 21589, Saudi Arabia; 2 Department of Cariology, Operative Dentistry, and Dental Public Health, School of Dentistry, Indiana University, 415 Lancing Street, Indianapolis, IN 46202, USA; flippert@iu.edu

**Keywords:** theobromine, caries, demineralization, fluoride, transverse microradiography

## Abstract

Developing artificial caries lesions with varying characteristics is needed to adequately study caries process in vitro. The objective of this study was to investigate artificial caries lesion characteristics after secondary demineralization protocol containing theobromine and fluoride. Sixty bovine enamel slabs (4 × 3 mm) were demineralized using a Carbopol-containing protocol for 6 days. A baseline area (2 × 3 mm) was protected with acid-resistant nail varnish, after which specimens were exposed for 24 h to a secondary demineralization protocol containing acetic acid plus one of four fluoride/theobromine combinations (*n* = 15): theobromine (50 or 200 ppm) and fluoride (0 or 1 ppm). Specimens were sectioned and analyzed using transverse microradiography for changes in mineral content, lesion depth, and surface layer mineralization. Data was analyzed using paired t-test and analysis of variance followed by Bonferroni test at 0.05 significance level. After secondary demineralization, fluoride-containing groups had significantly deeper lesions (*p* = 0.002 and 0.014) compared to the group with 0 ppm fluoride and 50 ppm theobromine. Mineral content and lesion depth were significantly different compared to baseline for all groups. Theobromine did not show an added effect on mineral uptake. Theobromine-containing groups exhibited particularly deep lesions with a more uniform mineral profile in the presence of fluoride.

## 1. Introduction

Dental caries is widely spread in many areas of the world and can be considered the most common chronic disease affecting the human population [1,2]. The use of preventive and non-surgical strategies is considered more beneficial and more cost-effective when managing this disease since caries can be reversed in its early stages [3,4]. Fluoride is one of the most effective agents utilized to control dental caries via decreasing the demineralization of teeth and enhancing the uptake of minerals by enamel and dentin [5,6,7]. However, depending on the lesions’ characteristics, fluoride might not be able to remineralize lesions with surface layers containing high mineral content since the lack of porosities in the surface layer could possibly hinder the passage of additional minerals [8,9].

Several animal studies have reported some anti-caries effects of chocolate [10,11]. Theobromine, a natural alkaloid extract from cocoa beans, can enhance the remineralization potential of fluoride and calcium-containing systems [12]. In laboratory studies, theobromine has been reported to decrease the dissolution of hydroxyapatite and form precipitates on the enamel surface [12,13]. Theobromine products have shown potential to decrease dentin hypersensitivity and enhance the rehardening of enamel lesions [14,15,16]. The synergistic effect of theobromine and fluoride could potentially enhance the overall remineralization power of de-/remineralization systems, leading to lesions with unique mineral distributions and profiles. The concentration of solutions can affect the remineralization behavior observed with artificially induced lesions in the presence or absence of fluoride. Furthermore, the effect of additional demineralization instances can affect the progression of these lesions.

The objective of this study was to investigate artificial caries lesion characteristics after a secondary demineralization challenge with an acetic acid solution containing fluoride and theobromine at various concentrations. This could help in creating caries lesions with unique mineral profiles different than the ones found in already-established lesions. The null hypotheses were a) that there are no differences in established lesions’ characteristics after secondary demineralization protocol between groups and b) there are no changes in lesions’ characteristics after secondary demineralization compared to baseline parameters.

## 2. Results

Normality and variance homogeneity assumptions were not violated (*p* > 0.05) necessitating a parametric analysis. Means and standard deviations of study parameters in addition to results of statistical analyses are included in Table 1. There was no significant difference between all experimental groups before secondary demineralization in ΔZ (*p* = 0.512), L (*p* = 0.882), and SZ_max_ (*p* = 0.875).

**Figure 1:**
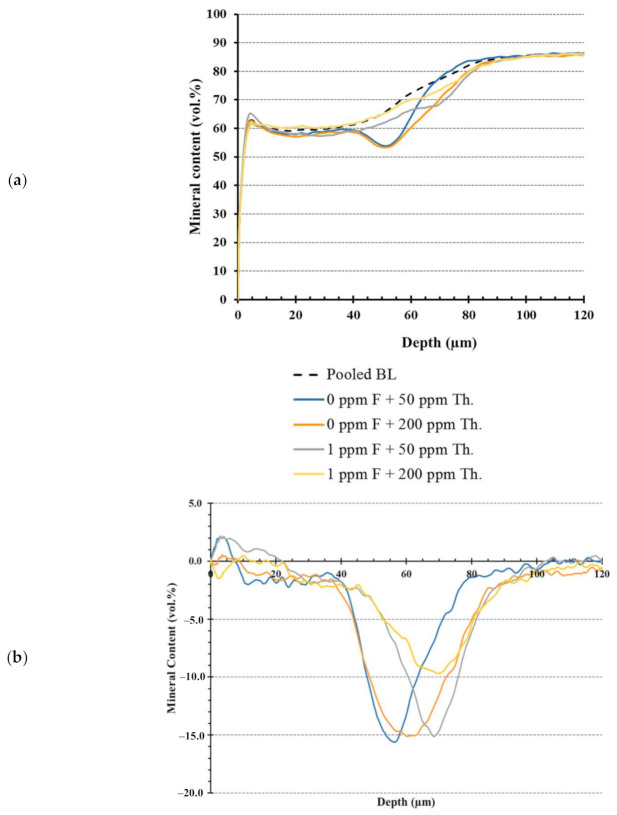
Graphs showing the mineral distribution for the study groups created by averaging values from all specimens in each group: (**a**) overall mineral distribution profiles for pooled specimens before secondary demineralization at baseline (dotted black line) compared to individual groups (solid lines) after secondary demineralization protocol and (**b**) changes in mineral content for the four experimental groups after respective secondary demineralization protocol with varying fluoride(F)/theobromine (Th.) concentrations.

After secondary demineralization, for all three lesion parameters the “theobromine” and the “theobromine.fluoride” interaction terms were non-significant (*p* > 0.05). For “fluoride” term, there was a significant difference (*p* = 0.002) between study groups in relation to lesion depth. Groups C and D had significantly (*p* = 0.002 and 0.014, respectively) deeper lesions than group A (Figure 1). No significant differences were found between groups regarding ΔΔZ (*p* = 0.319) or ΔSZ_max_ (*p* = 0.629).

**Figure 2:**
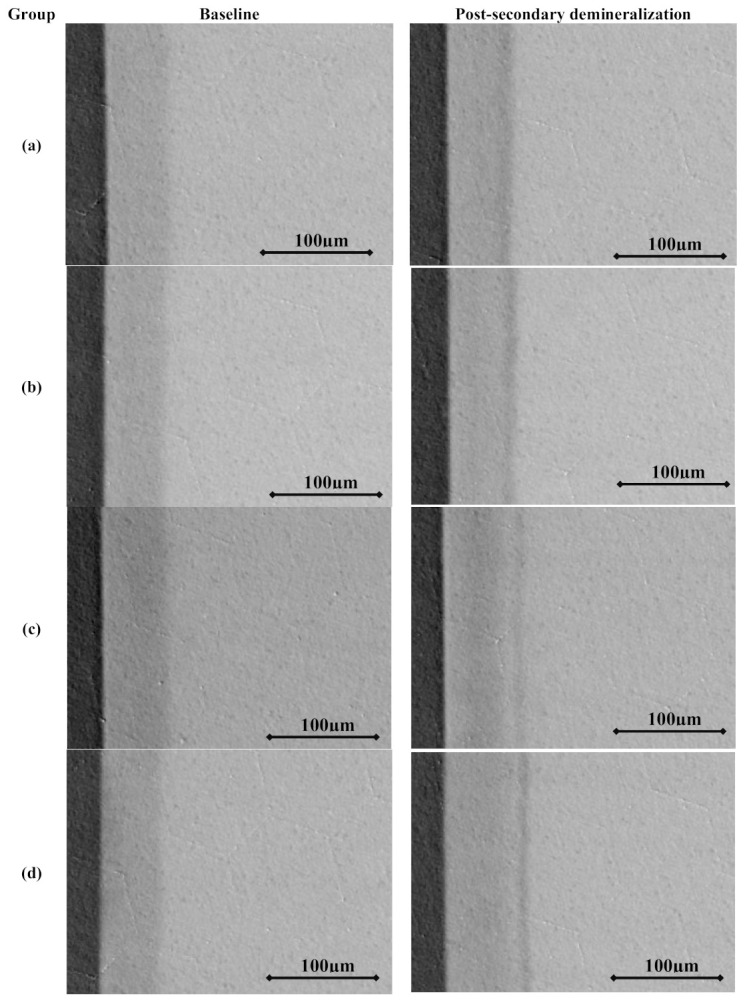
Transverse microradiographs of specimens from different study groups before and after secondary demineralization: (**a**) 0 ppm fluoride + 50 ppm theobromine, (**b**) 0 ppm fluoride + 200 ppm theobromine, (**c**) 1 ppm fluoride + 50 ppm theobromine, and (**d**) 1 ppm fluoride + 200 ppm theobromine.

Regarding within-group study parameters, there were significant differences in ΔΔZ and ΔL for all experimental groups before and after secondary demineralization (Figure 2). Overall, there was more mineral loss and deeper lesions after secondary demineralization without significant changes in surface layer mineralization (*p* ≥ 0.114).

## 3. Materials and Methods

### 3.1. Specimen Preparation and Initial Demineralization

Sixty enamel slabs (4 × 3 mm) were obtained from bovine teeth stored in 0.1% thymol solution. Both the anatomical (enamel) surface as well as the underside of all slabs were sequentially ground flat using silicon carbide grinding papers (RotoPol31/RotoForce 4 polishing unit; Struers, Cleveland, Ohio, USA). Slabs were assessed using a stereomicroscope (SMZ 1500, Nikon Instruments Inc., Melville, NY, USA) for cracks, hypomineralization areas, and other flaws. Prepared specimens were stored at 4 °C and 100% relative humidity until further use.

For the initial demineralization, bovine slabs were mounted on plastic rods and demineralized using a Carbopol protocol containing 50 mM lactic acid and 0.2% *w*/*v* Carbopol 907 at pH 5.0 (50% saturated with respect to hydroxyapatite), as previously described by White [17]. Specimens were demineralized for 6 days (10 mL/specimen), after which they were rinsed with deionized water and stored until further use.

### 3.2. Secondary Demineralization

Before secondary demineralization, a baseline area (2 × 3 mm) on the enamel surface was covered using acid-resistant nail varnish (Advanced Hard As Nails Nail Polish Natural, Sally Hansen Inc., Los Angeles, CA, USA) to protect the lesions created by the initial demineralization step. To test the possible changes on the mineral profiles of the established lesions created by the Carbopol protocol, secondary demineralization was accomplished by immersing the specimens at 37 °C for 24 h in a solution containing 50 mM acetic acid, 2.25 mM CaCl_2_·2H_2_O, 1.35 mM KH_2_PO_4,_ and 130 mM KCl, adjusted to pH 5.0 with KOH [18] plus one of the four fluoride/theobromine combinations: theobromine concentration (50 or 200 ppm) and fluoride concentration (0 or 1 ppm), as outlined in Table 1.

### 3.3. Transverse Microradiographic Analysis

After the secondary demineralization protocol was concluded, specimens were washed with deionized water and then sectioned using a hard tissue microtome (Hard Tissue Microtome, Series 1000 Deluxe; Silverstone-Taylor, SciFab, Lafayette, CO, USA) to give one 100-µm thick section of both initial and secondary demineralization areas from each specimen. Resulting sections were mounted on X-ray-sensitive plates (Microchrome Technology, Inc., San Jose, CA, USA) and subjected to X-ray radiation, accompanied by an aluminum step wedge to act as a standard. Images were analyzed with dedicated software (Inspektor TMR 2000, Ver.1.25) with the average mineral content of sound enamel set as 87% by volume [19,20]. Lesion characteristics included mineral content (ΔZ), lesion depth (L), and degree of surface layer mineralization (SZ_max_). Changes in lesions’ parameters between initial and secondary demineralization areas were also recorded, including changes in mineral content (ΔΔZ), changes in lesion depth (ΔL), and changes in surface layer mineralization (ΔSZ_max_), by subtracting baseline measurements of the initial lesions from values for each specimen after secondary demineralization protocol.

### 3.4. Statistical Analysis

Shapiro–Wilk test of normality as well as Levene test of variance homogeneity were conducted. Statistical testing was done using one-way analysis of variance (ANOVA) to compare baseline parameters before and after secondary demineralization across the four experimental groups, followed by Bonferroni post hoc analysis. Paired *t*-test was done to compare parameters before and after secondary demineralization protocol within each experimental group. To test the effect of fluoride and theobromine after secondary demineralization protocol, two-way ANOVA was used followed by Bonferroni post hoc analysis. All statistical analyses were conducted at 5% significance level using SPSS statistical software version 17 (IBM Corporation, Armonk, NY, USA).

## 4. Discussion

Dental caries is still a major public health concern in many countries in the world [1,2]. Utilizing preventive and remineralizing agents, such as fluoride, can be more beneficial owing to preserving the natural tooth structure [3,4,6]. However, sometimes the remineralization potential of fluoride can be reduced in lesions with highly mineralized surface zones [8]. In this circumstance, the addition of a supplementary remineralization agent, such as theobromine, can modulate the effect of fluoride producing lesions with different mineral profiles [12,13]. Furthermore, the combined effect of fluoride and theobromine can potentially result in the creation of incipient lesions with distinctive characteristics, especially if utilized on already-established lesions. Thus, the objective of this study was to investigate artificial caries lesion characteristics after secondary demineralization protocol containing fluoride and theobromine.

The demineralization protocol used in this investigation has been used previously to create incipient subsurface lesions with a mineral profile of L = 30–70 µm and SZ_max_ = 0.6–0.8 vol.% depending on the demineralization duration of the protocol [21]. Bovine teeth were used in this study since their dissolution behavior tends to be comparable to human teeth despite being easier to demineralize due to their different chemical composition and structure [22]. A detailed appraisal of the differences between human and bovine teeth was included in a recent review [23]. Transverse microradiography (TMR) is a verified test to quantify changes in mineral distribution. Although surface microhardness has been correlated previously with TMR [21], the subtle mineral distribution changes in deeper lesions necessitate a microradiographic analysis. One ppm fluoride was used in the secondary protocol since it has shown previously to affect the mineral uptake in remineralizing media. For theobromine, arbitrary concentrations of 50 and 200 ppm were used. Results from the current investigation will guide us in choosing future protocols with more granular modifications of the utilized concentrations of the active agents based on the resultant lesions’ mineral profiles.

The potential for theobromine to influence the mineralization behavior depends on the characteristic of the lesion as well as the methodology used. Lippert reported no added effect for theobromine in rehardening established lesions when combined with fluoride and strontium [24]. These findings are in agreement with results from the current investigation where no significant effect for theobromine was reported for ΔΔZ and ΔSZ_max_ between study groups. Still, in the absence of fluoride, using a higher theobromine concentration produced deeper lesions (group B). Further, even though the changes in mineral content in the fluoride/theobromine groups were similar (groups C and D), the lesion depths in these groups were significantly greater than the group with lower theobromine concentration (group A). This is in agreement with a previous study that showed that theobromine can affect the mineralization effect of fluoride, even though that report did not report microradiographic mineral profiles [12]. This could indicate that the active anti-caries potential of theobromine reported in previous animal studies might be due to an effect on microbial biofilms rather than a mechanistic effect on mineral uptake per se as speculated in another report [24].

Contrary to these findings, the report by Amaechi and collaborators indicates that theobromine has significant mineralization potential [12]. However, as stated above, this could be due to differences in initial lesions’ parameters. Amaechi et al. utilized hydroxyethyl cellulose lesions created in extracted human teeth, producing lesions that were shallower (~41 µm) than the presently employed Carbopol-lesions (~65 µm), in addition to significant differences in lesion mineral distribution profiles at baseline such as ΔZ values ranging between 680 and 815 vol.%. Further, their model contained pH cycling through the utilization of artificial saliva as a remineralization medium that contained additional minerals that could have affected the outcome. Additionally, the primary focus of the previous two studies was to depict the remineralization potential of theobromine in a more-mineralization focused protocol. In contrast, in the current investigation, the effect of theobromine on the progression of already-established Carbopol-created incipient lesions was the primary focus aiming to produce lesions with varying mineral distributions after the secondary demineralization regimen without the use of pH cycling steps. Portraying different mineral profiles of the resultant lesions can be beneficial when deeper lesions with gradual change in ΔΔZ throughout the lesions’ depth are targeted (Figure 1a). Fluoride in the present study produced deeper lesions with a steep curve of mineral profile starting around 50 µm depth. The presence of fluoride has caused an effect on mineral behavior leading to shallower yet more mineralized lesions.

The acetic acid secondary demineralization protocol was effective in further progressing the established lesions created by the Carbopol step in regards to both mineral content and lesion depth. However, it should be noted that no effect on the mineral content of the surface zone can be reported, indicating less ability for fluoride to affect the surface layer in the presence of theobromine, according to the studied conditions. As reported previously, the remineralization effect of fluoride in vitro depends on the type of the lesion [9]. Lesions with higher mineral content in the surface layer tend to remineralize less due to the presence of more minerals and lower porosity, preventing easy passage of minerals in deeper areas of the lesion [8].

Clear changes in mineral distributions of the study groups compared to pooled baseline lesion profile (Figure 1a) illustrate that different mineral profiles can be achieved by modulating the concentration of fluoride and theobromine in the secondary demineralization protocol. This is much apparent in Figure 1b, where lesion depth and mineral distributions are altered. It is interesting to see that group D (200 ppm theobromine and 1 ppm fluoride) had a lower mineral loss peak than the other groups, possibly indicating some protective effect for fluoride and theobromine. This effect might be further augmented if a higher theobromine concentration is to be used as will be investigated in the future. Creating incipient caries lesions with different mineral profiles can help researchers study caries lesions in multitudes of mineral profile distributions. The utilization of fluoride alone can reach its maximum potential since the remineralization power of fluoride can drive minerals leading to precipitation on the surface layer without allowing for mineral deposition in deeper zones of the lesion.

Contrary to other studies where there was an effect for the addition of fluoride [12,24], it should be noted that the presence of fluoride in the secondary demineralization protocol caused minor differences in the final mineral content of the lesions and possibly due to the short duration of the secondary protocol (24 h). Further studies are required to depict the effect on increasing the theobromine concentration, increasing the duration of secondary protocol, and the addition of pH cycling. Nonetheless, the resultant lesions have different mineral profiles compared to baseline parameters. Theobromine in the presence of fluoride produced lesions with more uniform mineral distribution throughout lesions’ depth compared to the low theobromine group. Still, all the resultant lesions have comparable mineral content in the surface layer ranging between 63 and 67 vol.%. It would be interesting to investigate the effect of the increase in the remineralizing power associated with higher fluoride concentrations past the 1 ppm utilized in the present investigation.

As with other laboratory investigations, this study had some limitations. First, the secondary demineralization protocol did not include pH cycling steps. This was done in order to isolate the effect of theobromine/fluoride combinations. A second limitation is the use of a single demineralization protocol, which created lesions that might not fully resemble incipient lesions. Still, variability of mineral characteristics of incipient lesions is high and it would be difficult to find one optimum demineralization model. However, future studies involving other demineralization protocols are justified in order to investigate the effect on lesions with more diverse mineral profiles. In addition, utilizing different concentrations of fluoride/theobromine and increasing the duration of the protocol are warranted.

The use of theobromine did not produce an added effect on mineral uptake. The tested fluoride-theobromine combination produced deeper lesions with a more uniform mineral distribution profile than theobromine alone; thus, we have rejected both of the null hypotheses. Still, the secondary demineralization protocol produced changes in both mineral content and lesion depth without affecting surface layer mineralization. This would beneficial in studying caries lesions with different profiles while maintaining mineral content in the surface zone.

## Figures and Tables

**Table 1:** Transverse microradiographic parameters before and after secondary demineralization protocol for experimental groups showing means, standard deviations (between parenthesis), and statistical analysis.

Group	Fluoride (ppm)	Theo-bromine (ppm)	*n*	Mineral Loss – ΔZ (vol.%)				Lesion Depth – L (µm)				Surface Zone Mineralization –SZ_max_ (vol.%)			
				Pre	Post	ΔΔZ	*p*-Value for Paired *t*-Test ^1^	Pre	Post	ΔL	*p*-Value for Paired *t*-Test ^1^	Pre	Post	ΔSZ_max_	*p*-Value for Paired *t*-Test ^1^
A	0	50	11	1607.3 (253.6)^A/a^	1995.5 (331.6)	−388.2 (306.1)^B/a^	0.002	65.4 (8.5)^A/a^	72.3 (9.9)	−6.9 (6.7)^B/a^	0.006	63.2 (4.4)^A/a^	63.8 (4.2)	−0.6 (4.3)^A/a^	0.677
B	0	200	14	1667.9 (280.2)^A/a^	2236.4 (477.3)	−568.6 (343.3)^B/a^	< 0.001	66.7 (9.3)^A/a^	80.2 (13.7)	−13.5 (7.8)^B/a,b^	< 0.001	64.3 (5.6)^A/a^	64.0 (6.9)	0.3 (6.4)^A/a^	0.857
C	1	50	15	1674.0 (318.5)^A/a^	2045.3 (244.6)	−371.3 (376.7)^B/a^	< 0.001	66.1 (8.8)^A/a^	85.4 (8.1)	−19.3 (9.7)^B/b^	< 0.001	64.9 (4.2)^A/a^	66.9 (4.5)	−2.0 (4.5)^A/a^	0.114
D	1	200	14	1506.4 (435.8)^A/a^	1875.7 (391.4)	−369.3 (271.1)^B/a^	< 0.001	63.7 (13.5)^A/a^	81.1 (13.0)	−17.3 (7.3)^B/b^	< 0.001	63.9 (6.9)^A/a^	65.5 (5.6)	−1.7 (5.1)^A/a^	0.242
*p*-value for ANOVA ^2^				0.516	-	0.319	-	0.882	-	0.002	-	0.875	-	0.629	-

^1^ Paired *t*-test between pre- and post-secondary demineralization values for a particular parameter at 0.05 significance level. Different upper-case letters indicate statistically significant difference (*p* < 0.05) between pre- and post- secondary demineralization for a particular parameter. ^2^ One-way analysis of variance for a particular parameter at pre-secondary demineralization or two-way analysis of variance for post-secondary demineralization between the four experimental groups at 0.05 significance level. Different lower-case letters indicate statistically significant difference (*p* < 0.05) between experimental groups for a particular parameter at pre- or post- secondary demineralization.

## Data Availability

The data presented in this study are available on request from the corresponding author.

## Abbreviations

TMR	Transverse microradiography
L	Lesion depth
ΔL	Changes in lesion depth
SZmax	Degree of surface layer mineralization
ΔSZmax	Changes in surface layer mineralization
ΔZ	Mineral content
ΔΔZ	Changes in mineral content
